# Sex, Gender and Age Differences in Treatment Allocation and Survival of Patients With Metastatic Pancreatic Cancer: A Nationwide Study

**DOI:** 10.3389/fonc.2022.839779

**Published:** 2022-03-24

**Authors:** Esther N. Pijnappel, Melinda Schuurman, Anna D. Wagner, Judith de Vos-Geelen, Lydia G. M. van der Geest, Jan-Willem B. de Groot, Bas Groot Koerkamp, Ignace H. J. T. de Hingh, Marjolein Y. V. Homs, Geert-Jan Creemers, Geert A. Cirkel, Hjalmar C. van Santvoort, Olivier R. Busch, Marc G. Besselink, Casper H.J. van Eijck, Johanna W. Wilmink, Hanneke W. M. van Laarhoven

**Affiliations:** ^1^ Department of Medical Oncology, Amsterdam UMC, University of Amsterdam, Cancer Center Amsterdam, Amsterdam, Netherlands; ^2^ Netherlands Cancer Registry, Netherlands Comprehensive Cancer Organisation (IKNL), Utrecht, Netherlands; ^3^ Department of Oncology, Lausanne University Hospital and University of Lausanne, Lausanne, Switzerland; ^4^ Department of Internal Medicine, Division of Medical Oncology, GROW–School for Oncology and Developmental Biology, Maastricht UMC+, Maastricht, Netherlands; ^5^ Department of Oncology, Isala Oncology Center, Zwolle, Netherlands; ^6^ Department of Surgery, Erasmus MC Cancer Institute, Rotterdam, Netherlands; ^7^ Department of Surgery, Catharina Hospital, Eindhoven, Netherlands; ^8^ Department of Medical Oncology, Erasmus Medical Center, Rotterdam, Netherlands; ^9^ Department of Medical Oncology, Catharina Hospital, Eindhoven, Netherlands; ^10^ Department of Medical Oncology, University Medical Center Utrecht, Utrecht University, Utrecht, Netherlands; ^11^ Department of Medical Oncology, Meander Medical Center, Amersfoort, Netherlands; ^12^ Department of Surgery, St Antonius Hospital, Nieuwegein, Netherlands; ^13^ Department of Surgery, University Medical Centre Utrecht, Utrecht, Netherlands; ^14^ Department of surgery, Amsterdam UMC, University of Amsterdam, Cancer Center Amsterdam, Amsterdam, Netherlands

**Keywords:** pancreatic neoplasms, pancreatic cancer, sex, gender identity, drug therapy, systemic treatment, palliative treatment

## Abstract

**Background:**

Biological sex, gender and age have an impact on the incidence and outcome in patients with metastatic pancreatic cancer. The aim of this study is to investigate whether biological sex, gender and age are associated with treatment allocation and overall survival (OS) of patients with metastatic pancreatic cancer in a nationwide cohort.

**Methods:**

Patients with synchronous metastatic pancreatic cancer diagnosed between 2015 and 2019 were selected from the Netherlands Cancer Registry (NCR). The association between biological sex and the probability of receiving systemic treatment were examined with multivariable logistic regression analyses. Kaplan Meier analyses with log-rank test were used to describe OS.

**Results:**

A total of 7470 patients with metastatic pancreatic cancer were included in this study. Fourty-eight percent of patients were women. Women received less often systemic treatment (26% vs. 28%, P=0.03), as compared to men. Multivariable logistic regression analyses with adjustment for confounders showed that women ≤55 years of age, received more often systemic treatment (OR 1.82, 95% CI 1.24-2.68) compared to men of the same age group. In contrast, women at >55 years of age had a comparable probability to receive systemic treatment compared to men of the same age groups. After adjustment for confounders, women had longer OS compared to men (HR 0.89, 95% CI 0.84-0.93).

**Conclusion:**

This study found that women in general had a lower probability of receiving systemic treatment compared to men, but this can mainly be explained by age differences. Women had better OS compared to men after adjustment for confounders.

## Introduction

Pancreatic cancer has a higher incidence in men than in women. In The Netherlands in 2019, the incidence of pancreatic cancer in absolute numbers for men was 1324 (52%) compared to 1245 for women (48%) (1–3). Many studies have reported on the predominance of pancreatic cancer diagnosis in men (1–5). Also, worse survival has been described for men suffering from pancreatic cancer (1–5).

Differences in incidence rates and outcome among women and men might be explained by biological (sex) and gender based-causes. These biological factors include sex differences in molecular and genetic subtypes (e.g. BRCA mutations). Gender-related factors are, for example, individual exposure to risk factors as tobacco and obesity (6–10). Also, gender may impact patient and physicians’ attitudes (11) and accessibility to health care.

Sex differences in cancer risk and survival have been described for multiple cancer types (12). Theoretically, sex differences in cancer survival may be attributed to differences in disease stage and/or (sub)-type at diagnosis, differences in biology of a given type of cancer of similar stage, differences in treatment allocation or differences in treatment effects.

Differences in treatment effects are classified in differences in pharmacokinetics and differences in pharmacodynamics (13, 14). However, little is known about the association between gender and the probability of receiving systemic treatment in metastatic pancreatic cancer. Examination of differences in treatment allocation and clinical characteristics of both men and women with metastatic pancreatic cancer might help to explain potential differences in outcome.

The aim of this study is to investigate patient characteristics, systemic treatment allocation and overall survival (OS) of women and men with metastatic pancreatic cancer in a nationwide cohort in general and also stratified for age ≤55 years, 56-64 years, 65-74 years and ≥75 years.

## Materials and methods

### Data collection

All patients diagnosed with synchronous metastatic pancreatic adenocarcinoma in The Netherlands between 2015 and 2019 were selected from the Netherlands Cancer Registry (NCR). In order to keep the patient population as homogenous as possible, we only included patients with metastatic disease. The NCR is a population-based registry containing data on all cancers in the Dutch population of over 17 million individuals. The database is directly linked with the nationwide network and registry of histology and cytopathology (PALGA), comprising all histologically confirmed cancer diagnoses. This registry, in combination with the National Registration of Hospital Care is a suitable representation of the metastatic pancreatic cancer patient population nationwide. Information about the patient (sex, age, performance status, previous cancer diagnosis, comorbidities), tumor (TNM-stage, tumor histology, location of primary tumor and metastases) and systemic treatment were identified from the hospital’s electronically health record system by trained registrars of the NCR. The main reason for deciding no cancer-directed treatment was also routinely registered in the NCR and categorized into comorbidity, social context, patient’s whish, short life expectancy, old age, extensive disease and other. Multiple metastases in one organ were defined as one metastatic site. Day to last follow-up was obtained by the annual linkage with data from the Municipal Personal Records Database, containing information on vital status and date of death from all Dutch inhabitants. These data were complete up to 1 February 2020. This study proposal was approved by the scientific committee of the Dutch Pancreatic Cancer Group (15). According to the Central Committee on Research involving Human Subjects, this type of study does not require approval from an ethics committee in the Netherlands. This study was designed in accoradance with the Strengthening the Reporting of Observational Studies in Epidemiology (STROBE) guidelines (16).

### Statistical Analysis

Data in this study were analyzed using SAS software (version 9.4, SAS institute, Cary, NC, USA). Patient and tumor characteristics were presented with means and standard deviations (SD) for continuous variables. Categorical variables were described with absolute numbers and percentages. Differences regarding patient and tumor characteristics between women and men were tested with chi-squared tests, or with Fisher’s exact tests when appropriate. The association between sex and the probability of receiving systemic treatment was examined with multivariable logistic regression analyses with adjustment for age, comorbidity, performance status, year of diagnosis and number of metastatic locations. OS was defined as the time interval from diagnosis until the end of follow-up or death. Kaplan Meier analyses with log-rank test were used to describe median OS and sex also stratified for age ≤55 years, 56-64 years, 65-74 years and ≥75 years because differences in outcome between patients of different sex in these age categories were expected based on the descriptives. The probability of a type-I error was set at 0.05.

## Results

### Patient Characteristics

A total of 7470 patients with metastatic pancreatic cancer were included in this study. Just under half of all patients were women (48%; [**Table 1**]). Median age was 71 years (IQR 63-78 years) and was slightly higher in women compared to men (72 vs. 70 years, P<0.001). Women had less comorbidities than men (P<0.001). Of all patients, 27% received systemic treatment and 73% best supportive care (BSC).

**Table 1:** Baseline characteristics of 7470 patients with metastatic pancreatic cancer stratified by sex.

Variable	All (n = 7470)	Men (n = 3884)	Women (n = 3586)	P value
Age years, median				
(IQR)	71 (63-78)	70 (63-77)	72 (64-79)	<0.001^a^
≤55	574 (8%)	326 (8%)	248 (7%)	<0.001^b^
56-64	1512 (20%)	831 (21%)	681 (19%)	
65-74	2726 (36%)	1460 (38%)	1266 (35%)	
≥75	2658 (36%)	1267 (33%)	1391 (39%)	
Tumor location, n (%)				
Head of pancreas	3089 (41%)	1598 (41%)	1491 (42%)	0.0098^b^
Body of pancreas	1274 (17%)	620 (16%)	654 (18%)	
Tail of pancreas	1870 (25%)	1027 (26%)	843 (24%)	
Overlapping sites	755 (10%)	381 (10%)	374 (10%)	
Pancreas NOS	482 (6%)	258 (7%)	224 (6%)	
Number of comorbidities, n (%)				
0	3047 (41%)	1441 (37%)	1606 (45%)	<0.0001^b^
1	2503 (34%)	1352 (35%)	1151 (32%)	
≥2	1376 (18%)	825 (21%)	551 (15%)	
Missing	544 (7%)	266 (7%)	278 (8%)	
Performance status, n (%)				
WHO 0-1	2630 (35%)	1411 (36%)	1219 (34%)	0.0017^b^
WHO 2	796 (11%)	444 (11%)	352 (10%)	
WHO 3-4	685 (9%)	362 (9%)	323 (9%)	
Unknown	3359 (45%)	1667 (43%)	1692 (47%)	
Year of diagnosis				
2015	1380 (18%)	746 (19%)	634 (18%)	0.1904^b^
2016	1533 (21%)	791 (20%)	742 (21%)	
2017	1485 (20%)	767 (20%)	718 (20%)	
2018	1522 (20%)	758 (20%)	764 (21%)	
2019	1550 (21%)	822 (21%)	728 (20%)	
Number of metastatic sites, n (%)				
1	4493 (60%)	2340 (60%)	2153 (60%)	0.854^b^
≥2	2977 (40%)	1544 (40%)	1433 (40%)	

n, number; IQR, interquartile range; NOS, not other specified; WHO, World Health Organization.

a Kruskal-Wallis test;

b Chi-Square test.

### Treatment

**Figure 1:**
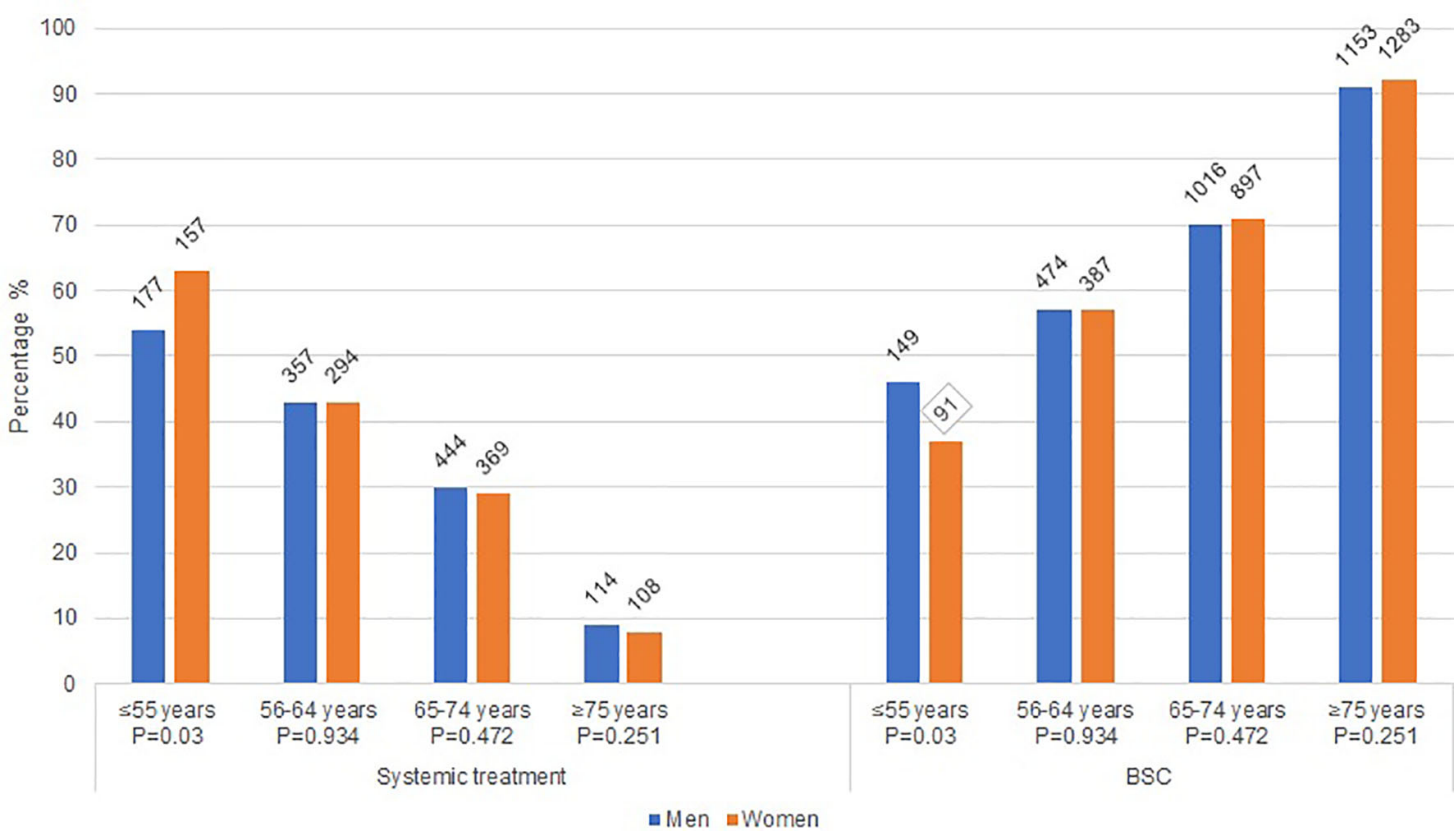
Treatment characteristics of women and men with metastatic pancreatic cancer. *BSC: best supportive care; P: Chi square p-value*.

Among all patients, women received less often systemic treatment as compared to men (26% vs. 28%, P=0.03). Differences were mainly seen in the younger age groups. **Figure 1** shows the treatment allocation (systemic treatment and BSC) of men and women by age category. Women aged ≤55 years received more often systemic treatment than men (p=0.03), whereas in the older age categories the allocation of systemic therapy did not differ. Furthermore, at younger age (≤55 years and 56-64 years) reasons for no administration of systemic treatment did not differ between women and men (P= 0.9952 and P=0.6195 [**Table 2**]). At higher age (65-74 years and ≥75 years) a significant difference in the reasons for not administering systemic treatment between women and men (P=0.0287 and P=0.0017) has been observed, with women choosing more often BSC.

**Table 2:** Reasons for no administration of systemic treatment in women and men with metastatic pancreatic cancer per age group.

Age groups	All patients		≤55 years		56-64 years		65-74 years		≥75 years	
Sex	Men	Women	Men	women	Men	Women	Men	Women	Men	Women
Patients not receiving systemic treatment (n)	2792	2658	149	91	474	387	1016	897	1153	1283
Main reason for not receiving systemic treatment:										
Wish patient (%)	33	38	30	27	38	40	36	43	30	35
Comorbidity/Performance status (%)	27	23	23	27	26	26	27	23	28	22
Progressive disease (%)	19	19	21	23	17	13	16	15	21	22
Death after diagnosis (%)	5	5	5	5	5	6	6	5	5	4
Age (%)	1	2							3	4
Situation at home (%)	0	0							0	
Other (%)	6	6	3	3	6	6	6	4	6	7
Missing (%)	8	15	17	13	9	9	8	32	7	6
Chi square p-value		0.0002		0.9952		0.6195		0.0287		0.0017

### Association of Biological Sex and the Probability of Receiving Systemic Treatment

Logistic regression showed that among all patients, women had a lower probability of receiving systemic treatment compared to men (adjusted odds ratio [OR] 0.89, 95% confidence interval [CI] 0.81-0.99). When we restricted our analyses to patients with a good performance status (0–1), the patients generally most suitable for systemic therapy, we did not find a statistically significant difference in the probability of receiving systemic treatment between women and men (OR 0.92, 95% CI 0.79-1.07). However, in patients with performance status 2 or higher we did find a statistically significant difference to the disadvantage of women (OR 0.89, 95% CI 0.81-0.98). The statistically significant difference between women and men observed in the total group of patients is therefore driven by performance status.

Multivariable logistic regression analyses, stratified by age category, showed that at ≤55 years of age, women were more likely to receive systemic treatment (OR 1.82, 95% CI 1.24-2.68 [**Table 3**]) as compared to men of the same age group. In the older age categories the probababilty to receive systemic treatment did not signifcantly differ between women and men (56-64 years OR women vs men) 0.99, 95% CI 0.80-1.24; and 65-74 years OR 0.93, 95% CI 0.76-1.10; and ≥75 years OR 0.85, 95% CI 0.63-1.13). When we restricted our analyses to patients with a good performance status (0–1), we found comparable results. At younger age ≤55 years, women had a higher probability of receiving systemic treatment compared to men (OR 1.83, 95% CI 1.02-3.29). Older women and men had no significantly different probability to receive systemic treatment (55-64 years OR (women vs men) 0.89, 95% CI 0.65-1.21; and 65-74 years OR 0.94, 95% CI 0.74-1.21; and ≥75 years OR 0.96. 95% CI 0.65-1.42).

**Table 3:** Multivariable logistic regression analyses for the probability of receiving systemic treatment in patients with metastatic pancreatic cancer stratified by age.

	≤55 years (n=574)			56-64 years (n=1512)			65-74 years (n=2726)			≥75 years (n=2658)		
	OR	95% CI	P value	OR	95% CI	P value	OR	95% CI	P value	OR	95% CI	P value
Sex												
Men	Reference			Reference			Reference			Reference		
Women	1.82	1.24-2.68	0.0025	0.99	0.80-1.24	0.942	0.93	0.76-1.10	0.385	0.85	0.63-1.13	0.260
Performance status												
WHO 0-1	Reference			Reference			Reference			Reference		
WHO 2	0.22	0.12-0.41	<0.001	0.48	0.33-0.69	<0.001	0.51	0.39-0.67	<0.0001	0.53	0.35-0.79	<0.0001
WHO 3-4	0.04	0.01-0.10	<0.001	0.07	0.04-0.14	<0.001	0.07	0.04-0.12	<0.0001	0.03	0.01-0.13	0.0021
Unknown	0.21	0.14-0.32	<0.001	0.29	0.23-0.37	<0.001	0.25	0.21-0.31	<0.0001	0.15	0.10-0.20	<0.0001
Number of comorbidities												
0	Reference			Reference			Reference			Reference		
1	1.03	0.65-1.63	0.905	0.80	0.63-1.03	0.0842	0.90	0.74-1.10	0.311	0.95	0.69-1.31	0.749
≥2	0.85	0.31-2.32	0.757	0.53	0.37-0.76	0.0007	0.72	0.56-0.93	0.0117	0.48	0.30-0.75	0.0015
Unknown	0.46	0.24-0.88	0.0187	0.79	0.51-1.24	0.305	0.67	0.46-0.97	0.0319	0.85	0.46-1.57	0.594
Number of metastatic sites												
1	Reference			Reference			Reference			Reference		
2 or more	0.76	0.52-1.10	0.147	1.04	0.83-1.29	0.761	1.06	0.89-1.27	0.497	0.77	0.56-1.04	0.0905
Year of diagnosis												
2015	Reference			Reference			Reference			Reference		
2016-2019	1.06	0.93-1.21	0.334	0.99	0.92-1.07	0.867	1.03	0.97-1.10	0.356	1.04	0.94-1.16	0.470

OR, odds ratio; 95% CI, 95% confidence interval; WHO, World Health Organization.

### Survival

**Figure 2:**
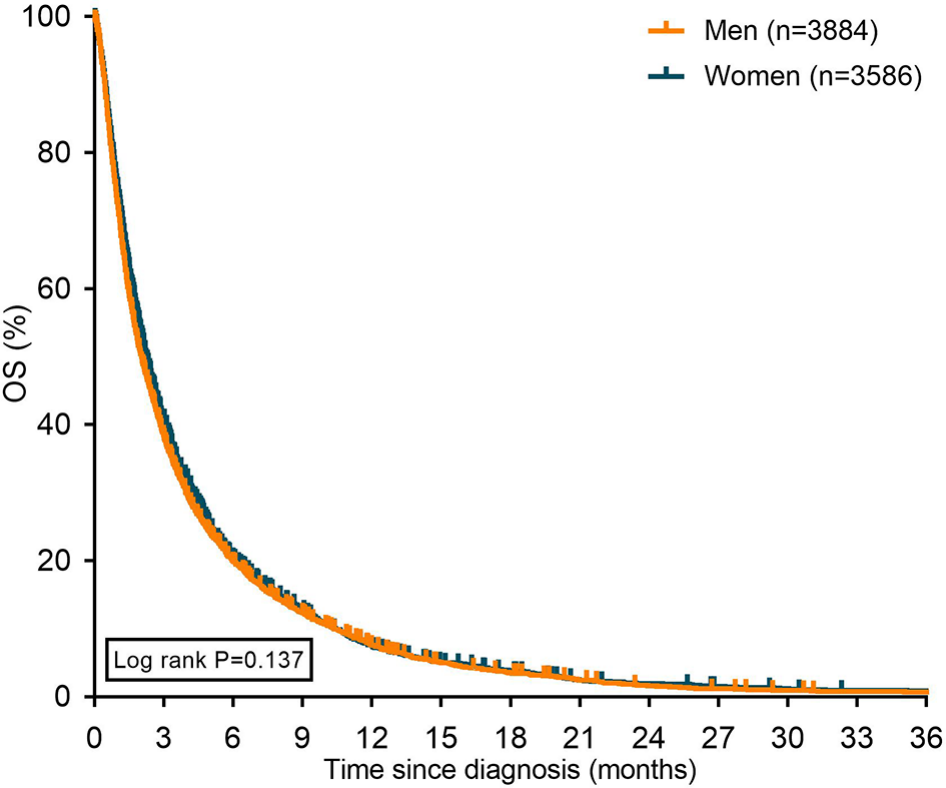
Kaplan Meier curves displaying overall survival in patients with metastatic pancreatic cancer stratified for sex. *OS: overall survival*.

Median OS of women with metastatic pancreatic cancer was 2.3 months and 2.1 months for men with metastatic pancreatic cancer (P=0.137 [**Figure 2**]).

**Figure 3:**
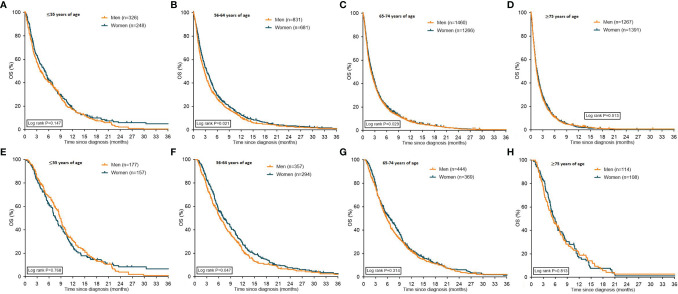
Kaplan Meier curves displaying overall survival in patients with metastatic pancreatic cancer stratified for sex. Graphs **(A–D)** depict all patients with pancreatic cancer stratified for sex, graphs **(E–H)** depict patients with pancreatic cancer who received systemic treatment stratified for sex. *OS: overall survival*.

In most age groups, women had (slightly) better median OS compared to men (**Figure 3**), except for the oldest age group (≥75 years of age) and in patients ≤55 years of age receiving systemic treatment.

In patients treated with BSC-only the median OS was only different between women and men in the age groups 56-64 and 65-74 years. Median OS in the age group ≤55 years was 1.8 months for women and 1.7 months for men (P=0.08). Women aged 56-64 years had a median OS of 1.8 months versus 1.5 months for older men (P=0.007). In the age group 65-74 years, women had a median OS of 1.7 months compared to 1.4 months for men (P=0.0007). In the age group ≥75 years, women had a median OS of 1.4 months versus 1.3 months for men (P=0.207).

Multivariable Cox propotional hazard analyses including all patients showed that women had a longer OS compared to men after adjustment for confounders (adjusted hazard ratio [HR] 0.89, 95% CI 0.84-0.93 [**Table 4**]). Increasing age and performance status, and metastatic sites all resulted in an increased risk of dying. Compared to tumors located in the head of the pancreas, patients with tumors in the body and tail had an increased risk of dying. Multivariable Cox proportional hazard analyses stratified for the different age groups (≤55, 56-64, 65-74 and ≥75 years of age) showed similar results. Women had a longer OS compared to men in all age groups. Increasing performance status and number of metastatic sites resulted both in an increased risk of dying in all age groups (**Table 4**).

**Table 4:** Multivariable Cox proportional hazard regression analyses for overall survival.

	All patients (n = 7470)		≤55 years (n = 574)		56-64 years (n = 1512)		65-74 years (n = 2726)		≥75 years (n = 2658)	
	HR (95% CI)	P value	HR (95% CI)	P value	HR (95% CI)	P value	HR (95% CI)	P value	HR (95% CI)	P value
Sex										
Men	Reference		Reference		Reference		Reference		Reference	
Women	0.89 (0.84-0.93) <.0001		0.79 (0.66-0.95) 0.0137		0.88 (0.79-0.98) 0.0171		0.85 (0.79-0.92) <.0001		0.93 (0.86-0.99) 0.0387	
Age			–		–		–		–	
≤55 years	Reference									
56-64 years	1.11 (1.00-1.23) 0.0450									
64-74 years	1.15 (1.04-1.27) 0.0054									
≥75 years	1.18 (1.07-1.31) 0.0012									
Performance status										
WHO 0-1	Reference		Reference		Reference		Reference		Reference	
WHO 2	1.37 (1.26-1.49) <.0001		1.63 (1.19-2.23) 0.0026		1.42 (1.18-1.72) 0.0002		1.31 (1.15-1.50) <.0001		1.41 (1.22-1.63) <.0001	
WHO 3-4	2.07 (1.89-2.27) <.0001		2.49 (1.69-3.67) <.0001		1.89 (1.54-2.31) <.0001		2.20 (1.90-2.55) <.0001		2.16 (1.85-2.52) <.0001	
Unknown	1.63 (1.54-1.72) <.0001		1.30 (1.05-1.62) 0.0159		1.58 (1.40-1.79) <.0001		1.52 (1.39-1.67) <.0001		1.87 (1.69-2.08) <.0001	
Number of comorbidities										
0	Reference		Reference		Reference		Reference		Reference	
1	1.01 (0.96-1.07) 0.6980		0.90 (0.72-1.13) 0.3530		1.01 (0.89-1.14) 0.8955		1.04 (0.94-1.14) 0.4590		1.00 (0.90-1.09) 0.8667	
≥2	1.02 (0.95-1.09) 0.5901		1.06 (0.65-1.72) 0.8269		0.90 (0.76-1.08) 0.2574		1.00 (0.90-1.12) 0.9610		1.07 (0.96-1.19) 0.2243	
Unknown	0.85 (0.77-0.93) 0.0005		1.13 (0.83-1.55) 0.4420		0.66 (0.54-0.82) 0.0002		0.93 (0.79-1.09) 0.3507		0.85 (0.72-1.00) 0.0546	
Number of metastatic sites										
1	Reference		Reference		Reference		Reference		Reference	
2 or more	1.30 (1.24-1.37) <.0001		1.34 (1.12-1.61) 0.0015		1.43 (1.28-1.60) <.0001		1.40 (1.29-1.51) <.0001		1.15 (1.06-1.25) 0.0011	
Year of diagnosis										
2015	Reference		Reference		Reference		Reference		Reference	
2016-2019	1.01 (0.99-1.03) 0.4017		0.95 (0.89-1.02) 0.1343		1.03 (0.99-1.07) 0.1202		0.99 (0.96-1.02) 0.5670		1.02 (0.99-1.06) 0.1193	
Systemic treatment										
No	Reference		Reference		Reference		Reference		Reference	
Yes	0.31 (0.29-0.33) <.0001		0.23 (0.19-0.29) <.0001		0.25 (0.22-0.28) <.0001		0.31 (0.28-0.34) <.0001		0.40 (0.34-0.47) <.0001	
Tumor location										
Head of pancreas	Reference		Reference		Reference		Reference		Reference	
Body of pancreas	1.14 (1.07-1.22) 0.0002		1.06 (0.82-1.38) 0.6561		1.11 (0.96-1.30) 0.1650		1.16 (1.04-1.30) 0.0076		1.34 (1.01-1.28) 0.0309	
Tail of pancreas	1.21 (1.14-1.29) <.0001		1.21 (0.96-1.52) 0.1045		1.17 (1.03-1.34) 0.0206		1.24 (1.12-1.37 <.0001		1.23 (1.11-1.36) <.0001	
Overlapping sites	1.27 (1.17-1.38) <.0001		1.36 (1.01-1.82) 0.0421		1.33 (1.10-1.59) 0.0025		1.16 (1.01-1.34) 0.0411		1.35 (1.18-1.54) <.0001	
Pancreas NOS	1.28 (1.16-1.42) <.0001		1.08 (0.72-1.62) 0.7240		1.26 (1.00-1.58) 0.0502		1.37 (1.17-1.60) <.0001		1.25 (1.05-1.47) 0.0100	

HR, hazard ratio; 95% CI, 95% confidence interval; WHO, World Health Organization; NOS, not other specified.

## Discussion

In this population-based study on sex and gender differences in patients with metastatic pancreatic adenocarcinoma, treatment use and survival differed between women and men. In general, women were slightly less often treated with systemic therapy compared to men. At a younger age (≤55 years), women more often received systemic treatment than men, but this difference disappeared at older age. Overall, after adjustment for confounding factors, women had a more favourable overall survival, however it should be mentioned that this statistically significant difference in survival between women has limited clinical relevance since the difference described is 0.3 months only. These results confirm the hypothesis that gender may influence treatment allocation and survival in patients with metastatic pancreatic cancer.

Treatment allocation not only affects a patient’s survival, but also the quality of life (17). Consequently, it is important to create awareness of the potential impact of gender stereotypes of caregivers on treatment decisions for each individual patient as they may compromise a patients’ access to care. To be able to understand these differences, it is important to make a distinction between gender based (behavioral and/or social) and sex based (tumor biology) aspects.

Gender based aspects that may contribute to the treatment allocation process include the preferences of the patient, social support and (unconscious) discrimination of the health care giver (18). Overall, only 27% of the patients in our study received systemic treatment with a median overall survival of 2.1-2.3 months. These outcomes are in line with other real-world studies on systemic treatment for patients with metastatic pancreatic cancer in The Netherlands (19–21). Gender has been proposed to be the most prominent predictor of a patients’ preference and may have an impact on treatment choices (22). Women tend to prefer BSC only more often compared to men (18, 23) – an observation, which is confirmed in our study. However, this does not explain our finding that younger women have a higher probability to receive systemic treatment. Also, the lack of differences in the older age groups are not explained, nor the fact that at younger age there was no difference in reasons for not starting systemic treatment. Overall, women had less comorbidities compared to men, which might be related to the higher probability for younger women to receive systemic treatment in our study. The family support of patients, e.g. marital status, plays a role in the treatment decision of cancer patients too (24). Married patients seem to choose active treatment more often and this trend has also been described for patients with pancreatic cancer (25, 26). Unfortunately, we did not have information on the marital status of the patients in our study. Since it is known that older women more often have a single status compared to younger women, this might explain why younger women were more likely to receive systemic treatment in our study compared to women of older age (25, 26). Another gender based factor that may affect treatment allocation is the possible bias of health care givers. Physicians are known to be susceptible to stereotypes and preconceptions (27, 28). For instance, single patients are offered treatment less often because of the assumption that there would not be enough support throughout the treatment trajectory (29). It is difficult to relate this possible bias of health care givers to our patient population. While patients preferences, marital status and unconscious bias of health care givers are factors with potential impact, it is currently not completely understood why younger women receive more often systemic treatment compared to men of the same age group.

A sex based effect that plays a role in the development of pancreatic cancer is the female sex hormone. Women are less likely to develop pancreatic cancer, and this is not fully explained by the exposure to the main risk factors cigarette smoking, high body mass index and diabetes mellitus (all gender based aspects), which are all more common in men (30–33). Studies showed that the female sex hormone estrogen decreases pancreatic cancer growth, which might explain why women have a lower risk to develop pancreatic cancer compared to men at younger ages but not at older ages (34–37). In our study, which focused on metastatic disease, we found a higher age at diagnosis in women. Maybe the drop in estrogen levels after menopause could be an additional explanation besides the fact that women live longer than men and therefore can be diagnosed with pancreatic cancer at an older age than men (38).

Moreover female sex hormone might have an impact on survival by a protective effect (39). The outcome of our study, with women having a better survival compared to men, cannot completely be explained by the difference in hormone levels, because we assume that the majority of women in our study was post-menopausal. However, post-menopausal women still have a different endocrine system compared to men. Another explanation might be the suggestion that the efficacy of systemic treatment may be different in women and men (13). Studies with various chemotherapeutic agents in different cancer types have shown treatment responses and survival rates in the advantage of women (40–43). However, in randomized studies on patients with pancreatic cancer the hazard ratios show the same treatment effect in women and men (44, 45) and our study did not show important differences in the population with all patients, therefore a difference in treatment effect in our population is unlikely. Our study showed that older women (>55 years) had the same probability to be treated with systemic therapy compared to men. This suggests differences in disease biology in men and women that might be responsible for the longer survival of women and warrants further investigation.

A limitation of this study is that the performance status was unknown in 45% of the patients, consequently less optimal adjustment for performance status in multivariable logistic regression analyses was possible. Second, data on toxicity were not available in our study, therefore it was not possible to describe potential differences between men and women in toxicity of systemic treatment. Third, since literature is unequivocal about the effect of social and family support on the treatment decision of oncological patients, it is unfortunate that we did not have any information about marital status or social support of the patients in our study. These factors and their impact on treatment decisions need further investigation. Although the findings in our study on the percentage of patients being treated with systemic treatment and pancreatic cancer diagnosis being more common in men than in women are in line with other European and American studies (46, 47), it might be difficult to generalize our findings to the rest of the (Western) world because ethnic differences may have an impact. Information on ethnicity is not captured in our study because this was not registered in the NCR. Fifth, age subgroups in the stratified analyses were small and might not have enough power to become statistically significant due to the groupsizes. Since the aim of this study was to provide insight in the systemic treatment allocation and survival between women and men, describing the specific systemic treatment regimen was beyond the scope of this study. However, it would be interesting to describe therapy schedules and dose density in future studies to give a more comprehensive overview of OS in relation to treatment. In addition, in order to interpret treatment allocation and OS in a more complete group of patients with pancreatic cancer, it would be important to add information of patients of all stages of the disease with a need for systemic treatment (e.g. locally advanced disease) in future studies.

In conclusion, the current study showed a statistically significant sex difference in survival in multivariable analyses, with women having a slightly better outcome. Since this difference in survival is 0.3 months only the clinical impact is limited. This study suggested that differences in survival might not always be fully explained by patient and treatment characteristics, disease biology might also play a role in the survival of patients with pancreatic cancer. To further personalize the treatment of these patients, it is important to understand the biological basis for sex differences while tailoring medical decisions to the patients’ wish and be aware of and avoiding gender stereotypes. Besides, it would be of interest to further investigate the difference seen between the age categories. We were not able to explain why the more frequent application of systemic therapy among females, disappeared at older ages.

## Data Availability Statement

The data underlying this article will be shared on reasonable request to the corresponding author.

## Ethics Statement

Ethical review and approval was not required for the study on human participants in accordance with the local legislation and institutional requirements. Written informed consent for participation was not required for this study in accordance with the national legislation and the institutional requirements.

## Author Contributions

Conceptualization: all authors. Data curation: EP, MS, LG Formal analysis: EP, MS. Investigation: EP, MS, AW, JW, HWMvL. Methodology: EP, MS, AW, LG, JW, HWMvL. Project administration: EP, JW, HWMvL. Supervision: MB, JW, HWMvL. Validation: JW, HWMvL. Visualization: EP, JW, HWMvL. Manuscript preparation: EP. Writing-original draft: EP, MS, AW, LG, JW, HWMvL. Writing—review and editing: all authors. All authors contributed to the article and approved the submitted version.

## Conflict of Interest

AW has received non-financial support from Roche and has received institutional support from Abbvie, Daichi Sankyo, IPSEN, Lily, Merck, MSD, Pierre Fabre, Servier. JV-G has received non-financial support from Servier, and has received institutional research funding from Servier. All outside the submitted work. JWW has served as a consultant for Shire, Servier and Celgene and reports grands from Servier, Halozyne, Novartis, Celgene, Astra Zeneca, Pfizer, Roche, Amgen and Merck. HWMvL has served as a consultant for BMS, Celgene, Lilly, Merck, Nordic, and Servier and has received unrestricted research funding from Bayer, BMS, Celgene, Lilly, Merck Serono, MSD, Nordic, Philips, Roche and Servier.

The remaining authors declare that the research was conducted in the absence of any commercial or financial relationships that could be construed as a potential conflict of interest.

## Publisher’s Note

All claims expressed in this article are solely those of the authors and do not necessarily represent those of their affiliated organizations, or those of the publisher, the editors and the reviewers. Any product that may be evaluated in this article, or claim that may be made by its manufacturer, is not guaranteed or endorsed by the publisher.
